# *Kingella kingae* KK247, an Atypical Pulsed-Field Gel Electrophoresis Clone A Strain

**DOI:** 10.1128/genomeA.01228-14

**Published:** 2014-11-26

**Authors:** Laetitia Rouli, Catherine Robert, Didier Raoult, Pablo Yagupsky

**Affiliations:** a Aix Marseille Université, URMITE, UM63, CNRS 7278, IRD 198, Institut National de la Sante et de la Recherche Medicale 1095, Marseille, France; b Soroka Medical Center, Ben-Gurion University of the Negev, Be’er-Sheva, Israel

## Abstract

*Kingella kingae* strain KK247 was isolated from an adult Israeli patient with endocarditis. It belongs to pulsed-field gel electrophoresis clone A, has a 2,113,021-bp genome, a 15,507-bp plasmid that carries genes encoding β-lactamases, and possesses 45 transposases, compared to the 5 detected in other *K. kingae* strains.

## GENOME ANNOUNCEMENT

*Kingella kingae* is being increasingly recognized as an important invasive pediatric pathogen (1). To date, 48 incomplete *K. kingae* genomes are available. *K. kingae* strain KK247 was isolated in 2006 from an adult Israeli patient with endocarditis. This strain grows as pinpoint colonies on blood agar and chocolate agar plates, fails to grow on Thayer-Martin medium, and shows adequate development on GC medium, suggesting a defect in the menadione metabolism. The organism belongs to pulsed-field gel electrophoresis clone A, which has been detected in the pharynges of 19 of 242 (7.8%) healthy carriers (2) but in only 2 of 181 (1.1%) Israeli patients with invasive *K. kingae* infections (strains KK245 and KK247), suggesting reduced virulence (3, 4). A microscopic examination of KK247 shows long and twisted chains of coccobacilli, indicating impaired cell separation. This small-colony variant phenotype has never been observed in clone A strains. Similar to all PFGE clone A isolates studied so far, strain KK247 produces a TEM-1 β lactamase (4).

*K. kingae* KK247 was sequenced by the Illumina MiSeq method. A total of 1,290,482 reads were obtained, and a *de novo* assembly was performed by the A5 pipeline (5). The median depth coverage was 123×, with an *N*_50_ of 88,578. At the end, we obtained 32 scaffolds, with 2 scaffolds corresponding to the plasmid. The KK247 genome is 2,113,021 bp long (G+C content, 46.6%), and it has 48 tRNAs, one rRNA operon, and 2,177 protein-coding genes. The plasmid is 15,507 bp long, with a G+C content of 44.1%, and carries genes encoding 23 proteins. Among these proteins, RAST annotated one TEM-1 β-lactamase, one tetracycline efflux protein (TetA), 4 hypothetical proteins, 3 mobile elements (i.e., transposases), 3 proteins implied in replication, 3 transcriptional regulators, and one streptomycin kinase (StrB). Using COG, we assigned categories to 14 proteins, 5 of which are implied in transcription (K category), 2 in replication (L), 1 in amino acid transport and metabolism (E), 1 in carbohydrate transport and metabolism (G), 1 in coenzyme transport and metabolism (H), and 2 in defense mechanisms (V) (the β-lactamase and the streptomycin kinase); 2 are poorly characterized (R).

A comparison of the COG (6) categories (83% of the proteins were annotated) showed that KK247 follows the same trend as other available *K. kingae* genomes and is more similar to the other two clone A strains analyzed (BB114 [a carriage strain] and KK245). The unique difference found is in the L category (replication, recombination, and repair) due to the large number of transposases in KK247 (45 instead of 5 in other strains). KK247 contains the same phage as BB114 and KK245 and had no clustered regularly interspaced short palindromic repeats (CRISPRs).

The proteins were determined by Prodigal (7), rRNAs by RNAmmer, and tRNAs by Aragorn (8). The plasmid was annotated by RAST (9).

### Nucleotide sequence accession number.

Strain KK247 and its plasmid have been deposited in EMBL under the project accession no. CCJT00000000.
